# Comparison of the Antiaging and Protective Properties of Plants from the *Apiaceae* Family

**DOI:** 10.1155/2020/5307614

**Published:** 2020-09-09

**Authors:** Nizioł-Łukaszewska Zofia, Zagórska-Dziok Martyna, Ziemlewska Aleksandra, Bujak Tomasz

**Affiliations:** Department of Technology of Cosmetic and Pharmaceutical Products, University of Information Technology and Management in Rzeszow, Kielnarowa 386a, 36-020 Tyczyn, Poland

## Abstract

Plant materials play a very significant role as components of products being used both for medicinal and cosmetic purposes. Due to the high content of active substances, they can play an important role as extracts with antioxidant, regenerative, and antiaging properties. The skin aging process depends on various pathological and physiological processes, among which the degradation of extracellular matrix biomolecules such as collagen and elastin, which significantly affect the maintenance of good skin condition, is very important. The secondary metabolites and plant extracts may have collagenase and elastase inhibitory activity. This activity is mainly due to the high content of a wide range of various biologically active compounds, such as polyphenols, which include, among others, flavonoids, phenolic acids, tocopherols, and tannins. The work involved a comprehensive assessment of the plant from *Apiaceae* family such as *Meum athamanticum L.*, *Centella asiatica* L., and *Aegopodium podagraria L.* extract as a multifunctional raw material. During study antioxidant properties, phenolic compounds and flavonoids content, effect on collagenase and elastase enzyme activity (antiaging effect), cytotoxic properties on skin cells (keratinocytes and fibroblasts), and cell migration capacity were analyzed. It has been shown that the highest antioxidant capacity can be observed for the extract of herb of *Aegopodium podagraria L*. When the concentration reached 5% all tested extracts had a positive effect on the cell proliferation of both keratinocytes and fibroblasts. It turned out that the most promising inhibitor of collagenase and elastase enzymes was the extract from *Aegopodium podagraria*, which inhibits the activity of both enzymes by over 70% in the concentration of 5% positively affecting the condition of skin cells.

## 1. Introduction

The aging process of the organism is caused by complex biochemical processes. Free radicals and reactive oxygen species (ROS) are one of the main factors inducing aging. They are particularly important in the skin aging process [1–3].

Free radicals and the oxidative stress effect on a number of changes in the structure and chemical composition of skin cells. Free radicals may cause the oxidation of lipids and proteins that build cell membranes, leading to their damage. After a cell membranes damage, they may cause DNA damage, finally leading to cell death. The effect of ROS on structural proteins of the skin (collagen and elastin) is extremely important in the skin aging process. As a result of the activity of free radicals, these proteins are damaged due to the activation of collagenase and elastase enzymes. Free radicals also may cause the degradation of hyaluronic acid (activation of hyaluronidases), which is responsible for the proper hydration of the skin and its firmness [1–6]. In addition to the effects of ROS on the skin aging process, free radicals may cause degradation of ceramides present in the skin, increasing transepidermal water loss from the epidermis (TEWL), and may cause oxidation of the lipid components of an intercellular cement, changing its structure and reducing barrier functions of the skin. The increase in TEWL and damage of the epidermal barrier due to ROS activities is one of the causes of skin irritations and allergies [7, 8].

Each organism is adapted to defend against ROS. Responsible for this are a number of enzymes naturally present in the skin and substances known as antioxidants. Under oxidative equilibrium, the free radicals produced in the body are neutralized by antioxidants. However, many factors such as a diet, lifestyle, and air pollution may disturb the oxidative balance, leading to oxidative stress. Under the oxidative stress conditions, the amount of substances capable of neutralizing free radicals is significantly lower than the amount of free radicals alone. It is therefore very important to derive exogenous antioxidants, for example, from a diet, cosmetic products, or pharmaceuticals [1–6].

The pharmaceutical, cosmetic, and food industries have been looking for substances with antioxidant activity for many years. Plant raw materials and extracts are excellent sources of natural antioxidants. The antioxidant capacity of plant extracts is mainly associated, for example, with content of phenolic, isoflavonoids, flavonoids, and anthocyanins. The widespread use of extracts in many industries is due to their multifunctionality. In addition to antioxidant activity, they contain a number of active substances that have a soothing, regenerating, or anti-inflammatory effect [4–6, 9–15].

During literature research, attention has been paid to plants from the *Apiaceae* family: *Meum athamanticum L.*, *Centella Asiatica* L., and *Aegopodium podagraria L*. For many years, these plants have been used in traditional medicine. *Aegopodium podagraria L.* is mainly used to treat urinary and kidney diseases. The main active ingredients of *Aegopodium podagraria L.* are proteins, carotenoids, carbohydrates, microelements (iron, copper, manganese, magnesium, potassium), polyacetylenes (falcarinol and falcarindiol), phenolic compounds (coumarins), flavonoids, caffeic and chlorogenic acid, and its derivatives and vitamins (C, E) [16–21]. *Centella asiatica* L. is a plant that has been used in Ayurvedic medicine for many years, mainly because of its regenerative, antibacterial, and memory-supporting properties, as well as in the treatment of respiratory and circulatory diseases. The data also indicate its anticancer effects. The main active ingredients are terpene saponins (asiaticoside, madecassoside, centelloside) and their derivatives. Other components of *Centella asiatica* L. include polyacetylene compounds, monoterpenes, sesquiterpenes, flavonoids, and carbohydrates. In addition, the plant is rich in vitamins E, A, K, and C [22–29]. *Meum athamanticum L.* is mainly used in the treatment of gastrointestinal and urinary tract diseases and liver regeneration. In addition, it has been shown to have antiviral activity (including HIV). Active ingredients include mainly saponins, terpenes, carbohydrates, phenolic compounds, and flavonoids [30, 31].

The aim of our research was to assess the effect of extracts from *Meum athamanticum L.*, *Centella Asiatica L.*, and *Aegopodium podagraria L* on viability of skin cells, fibroblasts, and keratinocytes *in vitro*. These cells were selected because this paper is an attempt to assess the potential use of extracts from this plant in pharmaceutical and cosmetic preparations. In addition, collagenase and elastase enzyme activity (antiaging effect) and cell migration capacity were determined. Due to the fact that oxidative stress plays an extremely important role in skin condition, this work also includes an assessment of the ability to scavenge free radicals and phenolic compounds and flavonoids content.

## 2. Materials and Methods

### 2.1. Plant Material and Extraction Procedure

*Aegopodium podagraria L*., *Centella asiatica L.*, and *Meum athamanticum L*. were obtained from the local herbalist. It was produced by FLOS, Natura Wita Sp. z o. o., and Nanga, Poland. Then, plant extracts were made separately from the herb of the *Apiaceae* family. Each sample was extracted using an 80 : 20 mixture of water and glycerin as a solvent. Extracts from plants of the *Apiaceae* family were obtained using an ultrasound-assisted extraction (UAE). UAE was performed according to the method described by Yang et al. in an ultrasonic bath (Digital Ultrasonic Cleaner) equipped with a time controller [32]. The mixtures were homogenized at room temperature for 48 minutes (6 cycles for 8 minutes). The obtained extracts were then collected and filtered three times through Whatman No. 10 filter paper using a vacuum pump. The extracts were stored in the dark at 4°C until further analysis.

### 2.2. Total Phenolic Content Determination

The assessment of total phenolic compounds content in extracts was analyzed spectrophotometrically using the Folin–Ciocalteu method with some modification [33]. This method depends on the reduction of Folin's reagent by phenols to a mixture of blue oxides which have a maximal absorption in the region of 740 nm. 300 *μ*l of tested extract sample (0.5–10%) and a standard solution of varying concentrations were mixed with 1500 *μ*l of 1 : 10 Folin–Ciocalteu reagent. The deionized water was used for dilution and a control sample. After incubation for 6 minutes in the dark at room temperature, 1200 *μ*l of 7,5% sodium carbonate solution was added to each sample followed by mixing and incubation for 2 h. The absorbance was read at *λ* = 740 nm on a spectrophotometer AquamateHelion (Thermo Scientific). The total phenolic compounds concentration was calculated from a gallic acid (GA) calibration curve (10-100 mg/ml). Data were expressed as gallic acid equivalents (GA)/g of extract averaged from three independent measurements.

### 2.3. Total Flavonoids Content Determination

The assessment of total flavonoid content in extracts was performed spectrophotometrically using aluminium nitrate nonahydrate by a method adopted by Matejić et al. with modifications [34]. According to this method, a reaction mixture was prepared containing 80% C_2_H_5_OH, 10% Al(NO_3_)_3_ × 9 H_2_O, and 1 M C_2_H_3_KO_2_. 2400 *μ*l of the previously prepared reaction mixture was mixed with 600 *μ*l of tested extract sample in various concentrations (0.5-10%). The deionized water was used for dilution and a control sample. After incubation for 40 min at room temperature, the absorbance was measured at *λ* = 415 nm with UV/VIS spectrophotometer AquamateHelion (Thermo Scientific). Quercetin was used as a standard for calibration curve, and the results were expressed as Quercetin equivalents (Qu/g) of extract averaged from three independent measurements.

### 2.4. DPPH Radical Scavenging Assay

The stable 1,1-diphenyl-2-picryl hydrazyl radical (DPPH) was used for the determination of free radical-scavenging activity of the tested plant extracts. This method was described by Brand-Williams et al. [35]. 33 *μ*l of tested plant samples at various concentrations (0.5-10%) were added to 167 *μ*l methanol solution of DPPH (4 mM) in a 96-well plate. The mixture was shaken vigorously and absorbance was recorded at *λ* = 516 nm with UV/VIS by using a spectrophotometer Filter Max 5 (Thermo Scientific). Measurements were carried out in triplicate for each sample. DPPH solution mixed with an equal volume of distilled water was used as a control. The scavenging activity on the DPPH radical was expressed as the inhibition percentage using the following equation:

(1) $$\begin{array}{c} \%\text{DPPH}•\text{scavenging}=\frac{\text{Abs control}-\text{Abs sample}}{\text{Abs control}}\text{ x }100\%, \end{array}$$

where Abs control is the absorbance of the control sample (containing all reagents except the test extract or standard), and Abs sample is the absorbance of the test extract or standard.

### 2.5. Cell Culture

HaCaT (normal human keratinocytes were purchased from CLS Cell Lines Service (Germany) was obtained from the American Type Culture Collection (Manassas, VA 20108, USA). HaCaT cells were maintained in a DMEM (Dulbecco's modified essential medium, Gibco) with L-glutamine, supplemented with 10% (vol/vol) FBS (fetal bovine serum, Gibco), and 1% (vol/vol) antibiotics (100 U/ml penicillin and 1000 *μ*g/ml streptomycin, Gibco). BJ cells (fibroblasts, ATCC®CRL-2522™) used in the study were obtained from the American Type Culture Collection (Manassas, VA 20108, USA). Fibroblasts were maintained in a MEM (Minimum Essential Medium, Gibco) contains Earle's salt and L-glutamine, supplemented with 5% (vol/vol) FBS (fetal bovine serum, Gibco), and 1% (vol/vol) antibiotics (100 U/ml penicillin and 1000 *μ*g/ml streptomycin, Gibco). All cultured cells were kept at 37°C in a humidified atmosphere of 95% air and 5% of carbon dioxide (CO_2_). When the cells reached confluence, the culture medium was removed from the flask (VWR), and cells were rinsed two times with sterile PBS (Phosphate-Buffered Saline, Gibco). The confluent layer was trypsinized using Trypsin/EDTA (Gibco) and then resuspended in fresh medium.

#### 2.5.1. Cell Viability Assay

Cell growth was measured using the neutral red dye (Sigma Aldrich). This assay is based on the initial protocol described by Borenfreund et al. (1984) and determines the accumulation of the neutral red dye in the lysosomes of viable, uninjured cells. Cells were placed in 96-well plates at a density of 1 × 10^4^ cells/well with fresh medium. After 24 h of preculture, the medium was aspirated, and varying concentrations 0,5% to 10% of extracts were added into each well and cultured for another 24 h. The control group was unexposed cells. Following exposure to leaves and tuber extracts, cells were incubated for 2 h with neutral red dye. After this, cells were washed with Phosphate Buffered Saline (PBS), and then added 150 *μ*l destain solution (EtOH/AcCOOH/H_2_O_2,_50%/1%/49%) per well, followed by gentle shaking for 10 min, until the neutral red has been extracted from the cells and has formed a homogeneous solution. Neutral red dye uptake was determined by measuring the optical density (OD) of the eluted dye at 540 nm in a microtiter plate reader spectrophotometer FilterMax F5 (Thermo Fisher). The experiments were performed in triplicates for each extract concentration and presented as percentage of control values (100%) [36].

The resazurin sodium salt (Alamar Blue) (Sigma, R7017) was used to assess cell viability. HaCaT and fibroblast cells were seeded in transparent 96-well plates and exposed to different extract concentration ranging from 0.5% to 10% 24 h. The control group was unexposed cells. After exposure, the resazurin solution was transferred into the plates for a final volume 250 *μ*l/well and final concentration of 60 *μ*M resazurin and incubated for 2 hours at 37°C. The absorbance was measured at the wavelength *λ* = 570 nm using a microplate reader (FilterMax F5, Thermo Fisher). The experiments were performed in triplicates for each extract concentration. Results were expressed as percentage cell viability versus the control (100%).

### 2.6. Determination of Anticollagenase Activity

The ability of the tested plant extracts to inhibit collagenase activity was analyzed using a fluorometric Collagenase Inhibitor Screening Kit (Abcam, ab211108). Extracts of all three tested plants in concentrations of 0.5 and 5% were used for the analysis. Samples were prepared for analysis in a 96-well plate with a clear flat bottom. In the first step, collagenase was dissolved in Collagenase Assay Buffer (CAB). The test samples were prepared by mixing the tested extract with collagenase and CAB. Inhibitor control samples were prepared by mixing inhibitor ((1,10)-Phenanthroline (80 mM)) with diluted collagenase and CAB buffer. Enzyme control wells were prepared by mixing diluted collagenase with CAB. The CAB buffer was used as background control. The samples were incubated for 15 minutes at room temperature. In the meantime, a reaction mixture was prepared by mixing the collagenase substrate with CAB. In the next step, the reaction mixture was added to the prepared samples and mixed thoroughly. The fluorescence was immediately measured with an excitation wavelength of 490 nm and emission 520 nm using a microplate reader (FilterMax F5, Thermo Fisher). The measurement was made in kinetic mode, for 60 minutes at 37°C. All samples were prepared in duplicate according to the manufacturer's instructions. The ability to inhibit collagenase activity by the analyzed samples was calculated from the equation:

(2) $$\begin{array}{c} \%\text{relative inhibition}=\frac{\text{enzyme control}-\text{sample}}{\text{enzyme control}}*100. \end{array}$$

### 2.7. Determination of Antielastase Activity

Fluorometric Neutrophil Elastase Inhibitor Screening Kit (Abcam, ab118971) was used to determine the ability of the extracts to inhibit the activity of the neutrophil elastase (NE) enzyme. Extracts of all three tested plants in concentrations of 0.5 and 5% were used for the analysis. Samples were prepared for analysis in 96-well black plates (clear bottoms) for fluorometric assay, and the analysis was performed according to the manufacturer's instructions. Briefly, neutrophil elastase enzyme solutions, NE substrate, and inhibitor control (SPCK) were prepared as initially as recommended. Then, diluted NE solution was added to all wells. Test samples, inhibitor control, and enzyme control (Assay Buffer) were applied to subsequent wells. All samples were prepared in duplicate according to the manufacturer's instructions. The samples were mixed thoroughly on a shaker and the plate incubated at 37°C for 5 minutes. The fluorometric reaction mix was then prepared by mixing Assay Buffer and substrate. The prepared reaction mixture was added to each sample and mixed thoroughly. The fluorescence was immediately measured with an excitation wavelength of 400 nm and emission 505 nm using a microplate reader (FilterMax F5, Thermo Fisher). The measurement was made in kinetic mode, for 30 minutes at 37°C protected from light. The ability to inhibit neutrophil elastase activity by the analyzed samples was calculated from the equation:

(3) $$\begin{array}{c} \%\text{relative activity}=\frac{∆\text{RFU test inhibitor}}{∆\text{RFU enzyme control}}*100. \end{array}$$

### 2.8. Scratch Wound Assay

In order to assess the possibility of stimulating the migration of keratinocytes and fibroblasts by extracts from *Meum athamanticum*, *Centella asiatica* L., and *Aegopodium podagraria*, a scratch test was carried out. For this purpose, cells were cultured in DMEM (Modified Dulbecco's Essential Medium, Corning) with L-glutamine, 4.5 g/l glucose, sodium pyruvate. Medium was supplemented with 10% FBS (fetal bovine serum, Gibco) and 1% antibiotics (100 U/ml penicillin and 1000 *μ*g/ml streptomycin, Gibco). Cells were cultivated in an incubator at 37°C in a humid atmosphere of 95% air and 5% carbon dioxide (CO2). After the cell monolayer reached the appropriate confluence, the cell layer was carefully scratched with a 10 *μ*l pipette tip. The cells were then treated with water-glycerin extracts of *M. athamanticum*, *C. asiatica*, and *A. podagraria* at concentrations of 0.5, 1, 2.5, 5, and 10%. The extracts were dissolved in an analogous medium that was used during cell culture, but with a reduced FBS content (1%). After 24 hours incubation, photos of cells treated with the tested extracts were taken under a magnification of ×10 under a microscope.

### 2.9. Statistical Analysis

Each value is the mean of three replicates. Obtained values were presented as mean ± SD. Significant differences between obtained values were analyzed using GraphPad Prism 5.0 software using One-way ANOVA and Tukey's test. The differences were considered significant when *p* < 0.05.

## 3. Results

Polyphenolic compounds such as phenolic acids, flavonoids, and tannins are commonly found in plant extracts from the *Apiaceae* family. Topical applications of cosmetic formulations containing phenolic compounds can reduce the causes and effects of skin aging, skin diseases, and damage [37]. Moreover, phenols and polyphenolic compounds, such as flavonoids, are widely found in cosmetic and pharmaceutical products derived from plant sources, and they have been shown to possess significant antioxidant activities [38]. Many authors have shown that *Aegopedium podagraria L*. contains polyphenols, e.g., phenolic acid and flavonoid, especially polyacetylenes (falcarinol and falcarindiol) [17, 18, 38–42]. Jakubczyk et al. showed that the total content of polyphenols in the ethanolic extracts from different parts of *A. podagraria L*. depends on the morphological part of the plant and the temperature of extraction. The highest total polyphenol content was recorded for flower extracts, the lowest for seeds extracts [43]. Chromatographic analysis has shown that leaves of this plant are characterized by a high content of lipophilic antioxidants including carotenoids, chlorophylls, and tocopherols. It was shown that the leaves had a high concentration of the xanthophylls lutein, neoxanthin, and VAZ (the total amount of violaxanthin, astaxanthin, and zeaxanthin) and total carotenoids, slightly lower than the nettle [18, 44]. According to the data [45, 46], hydroxycinnamic acids, ferulic acid, and chlorogenic acid are also present in A. *podagraria L*. raw material.

*Centella asiatica L.* is well known to have a high antioxidant activity [47]. Antioxidant activity of *C. asiatica L.* is comparable to the activity of rosemary and sage and has very good potential to be explored as a source of natural antioxidants [48]. Extract of *Centella asiatica L*. demonstrated high free radical scavenging activity of 83% inhibition at a concentration of 1 mg/ml. It was found that the inhibition effect of the free radical scavenging activity decreased in the order of green tea > vitamin C > Centella > grape seed extract. *C. asiatica L.* extract showed comparable activity with grape seed extract which is stated to be a powerful antioxidant due to its proanthocyanidin content [49]. Chromatographic analysis has shown a significant presence of the vitamin C, B1, B2, niacin, carotene, and vitamin A [49–51]. It was also reported to contain flavonoids, 3-glucosyl quercetin, 3-glucosyl kaempferol, and 7-glucosyl kaempferol. Apart from these, two new flavonoids named castilliferol 1 and castillicetin 2 have been isolated from the whole plant recently. Presence of several flavonoid derivatives such as quercetin, kaempferol, patuletin, rutin, apigenin, castilliferol castillicetin, and myricetin has been reported in C. asiatica [52, 53].

*Meum athamanticum L*. is also well known to have high antioxidant activity. Active ingredients include mainly essential oil, saponins, phenolic compounds, and flavonoids [30, 54–56]. Some of the components isolated from the extracts of *M. athamanticum* were cinnamic acid esters (methyl ferulate, methyl caffeate, quinic acid, and feruloyl quinic acid), phthalide derivatives (ligustilide, butylidenephthalide, and butylphthalide), and sterol derivatives (cetyl alcohol and sitosterol) [57–59].

**Figure 1:**
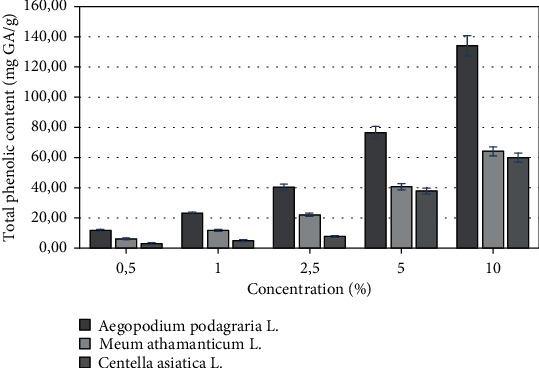
Total phenolic content of various concentrations of *A. podagraria L.*, *M. athamanticum L.*, and *C. asiatica L.* of water-glycerine extracts. Values are mean of three replicate determinations (*n* = 3) ± SD.

**Figure 2:**
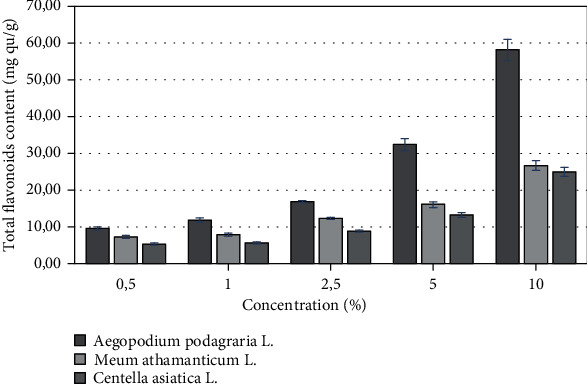
Total flavonoids content of various concentrations of *A. podagraria L*., *M. athamanticum L.*, and *C. asiatica L*. of water-glycerine extracts. Values are mean of three replicate determinations (*n* = 3) ± SD.

The total phenolic content of the *Apiaceae* family extracts was estimated by Folin–Ciocalteu assay and expressed in gallic acid equivalents, and it was calculated from the linear regression equation of standard curve (*y* = 0.0192*x* + 0.0652, *R*^2^ = 0, 9971). The total flavonoid content of the *Apiaceae* family extracts was determined via aluminium nitrate nonahydrate spectrophotometric method, and it was calculated from the linear regression equation of standard curve of quercetin (*y* = 0.0083*x* + 0.004, *R*^2^ = 0, 9906). The results showed (Figures 1 and 2) that the highest content of phenolic compounds as well as flavonoids among representatives of plants of the *Apiaceae* family was found in *Aegopodium podagraria L.* extract. At the highest tested concentration 10%, the content of phenolic compounds and flavonoids in the water-glycerin extract was 134 mg and 58 mg GA/g. It was noted that the total phenols and flavonoids content increased with concentration. In this study, the total phenolic compounds of the various extracts from commonly known plants of *Apiaceae* family were found to be in the ranges of 59,96–134.03 mg/g (as gallic acid equivalent) for the concentration of extracts 10%, and they could be largely responsible for the antioxidant activity of the *Apiaceae* family extracts.

The DPPH assay is based on the ability of 2,2-diphenyl-1-picryl-hydrazyl (DPPH), a stable free radical, to change color in the presence of antioxidants. The odd electron in the DPPH radical is responsible for the absorbance at 517 nm, and also for the visible deep purple color. When DPPH accepts an electron donated by an antioxidant compound, the DPPH changes color from purple to yellow which can be quantitatively measured from the changes in absorbance. Antioxidants can decrease the oxidative damage directly or indirectly. Direct action is mainly associated with reacting with free radicals, whereas the indirect action consists in inhibiting the activity or expression of free radical generating enzymes or increasing the activity or expression of intracellular antioxidant enzymes. DPPH radical scavenging assay is one of the most widely used methods for screening of antioxidant activity of plant extracts [60].

**Figure 3:**
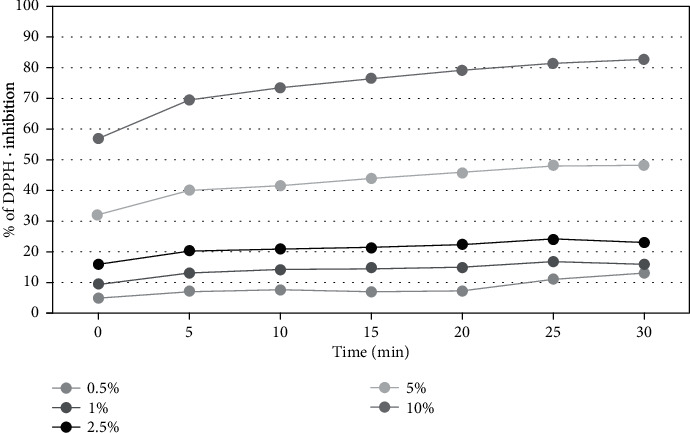
Kinetics of the absorbance changes in DPPH∙ solutions in the presence of various concentrations of water-glycerine extract of *A. podagraria L*. Values are mean of three replicate determinations (*n* = 3).

**Figure 4:**
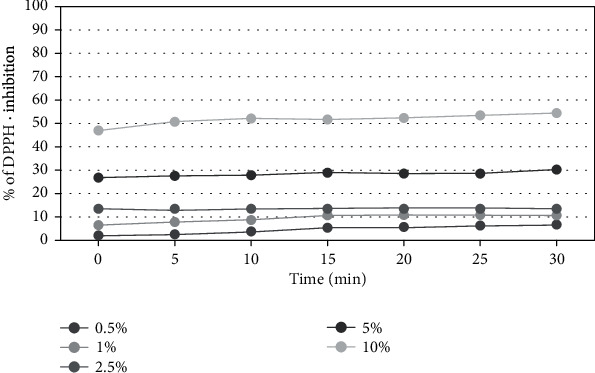
Kinetics of the absorbance changes in DPPH∙ solutions in the presence of various concentrations of water-glycerine extract of *M. athamanticum L*. Values are mean of three replicate determinations (*n* = 3).

**Figure 5:**
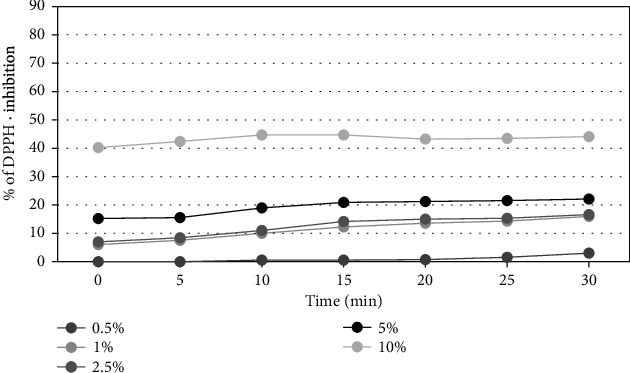
Kinetics of the absorbance changes in DPPH∙ solutions in the presence of various concentrations of water-glycerine extract of *C. asiatica L*. Values are mean of three replicate determinations (*n* = 3).

The results of the assay for antioxidant activity are shown in Figures 3, 4, and 5.

In order to determine the antioxidant properties of the extracts tested, five different concentrations were used from 0.5% to 10%. Measurements were taken every five minutes for 30 minutes. The results reveal that the percentage inhibition of DPPH radical was increased with concentration. All tested extracts from members of *Asteraceae* family plants show a significant ability to scavenge free radicals in a concentration of 5 and 10%. The highest free radical scavenging activity was observed for the extract of herb of *Aegopodium podagraria L*. After 30 minutes of the measurement, the level of reduced DPPH • reached almost 83%, while at the lowest concentration (0.5%) the level of scavenged radicals was about 13%. In all analyzed raw materials, a correlation between the concentration and the antioxidant potential of the extracts was also observed—the higher the concentration was used, the higher force of free radicals reduction. In order to evaluate antioxidant activity, additionally, designated EC50 values were determined for the DPPH measurements for the three analyzed plants in the 30 minute of the measurement (Table 1).

The studies show that these extracts are a significant source of natural antioxidants, which might be helpful in preventing the progress of various oxidative damages. Zainol et al. showed that there is a strong correlation between antioxidant activity and phenolic compounds content (*r*2 = 0.90), suggesting that phenolic compounds are probably responsible for the antioxidative activities of *C. asiatica* [60]. Similarly, Christova-Bagdassarian et al., evaluating the antioxidant potential of five different plant extracts belonging to the *Apiaceae* family, concluded that the most prominent bioactive potential was observed in the plant extract with the highest content of phenolics and flavonoids [61]. Velioglu et al. also found that phenolic compounds were responsible for the antioxidative activity in some selected fruits, vegetables, and grains tested [62]. Phenolic compounds are also effective hydrogen donors, which make them good antioxidants [63]. Thus, the therapeutic properties of members of plants *Apiaceae* family may possibly be attributed to the phenolic compounds present. Despite several studies on antioxidative compounds and plant properties, further investigations are required to isolate and identify the antioxidant compounds present in the plant extracts.

The biological activity of herbs of *Aegopodium podagraria L.*, *Meum athamanticum L.*, and *Centella asiatica* L. was also investigated on cells as *in vitro* models. The cytotoxicity of tested extracts was assessed on HaCaT and BJ fibroblast cell lines using resazurin and neutral red method. Both types of cells were treated with various extract concentrations, ranging from 0,5% to 10% for 24 h. Cytotoxic effect was observed for extracts only at the highest analyzed concentration which was 10%, excluding *Aegopodium podagraria* L., where, in the highest concentrations, a slight increase in cell proliferation was noted. It was also observed that the analyzed extracts have less toxic effect on HaCaT cells compared to the BJ cell lines. The highest cytotoxicity was found in *Meum athamanticum L*. extract at a concentration of 10% in the case of the BJ cells.

**Figure 6:**
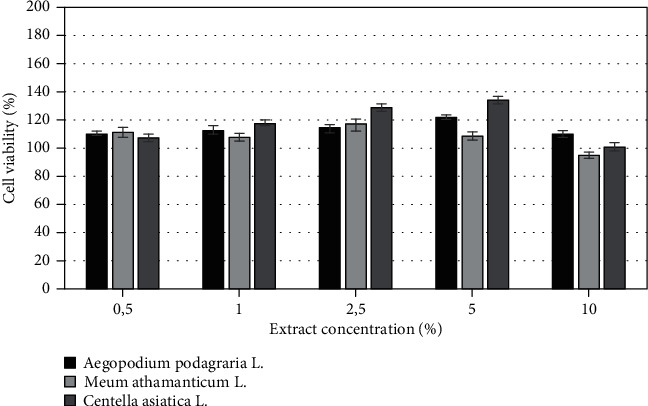
The effect of different concentrations of *Aegopodium podagraria L.*, *Meum athamanticum L.*, and *Centella asiatica L.* extracts (0,5%, 1%, 5%, 10%) on resazurin salt reduction in cultured keratinocytes after 24 h of exposure. Data are the mean ± SD of three independent experiments, each of which consists of three replicates per treatment group.

**Figure 7:**
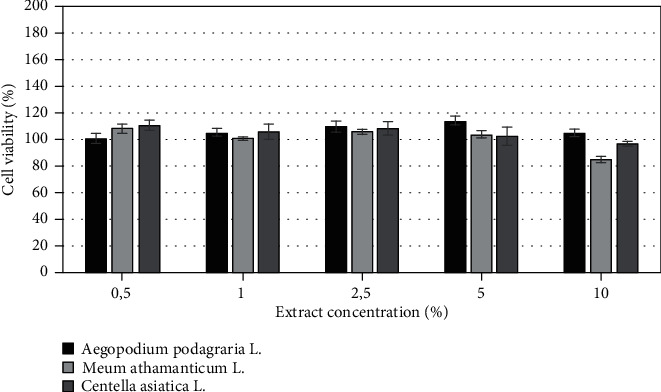
The effect of different concentrations of *Aegopodium podagraria L.*, *Meum athamanticum L.*, and *Centella asiatica L.* extracts (0,5%, 1%, 5%, 10%) on resazurin salt reduction in cultured BJ fibroblasts after 24 h of exposure. Data are the mean ± SD of three independent experiments, each of which consists of three replicates per treatment group.

**Figure 8:**
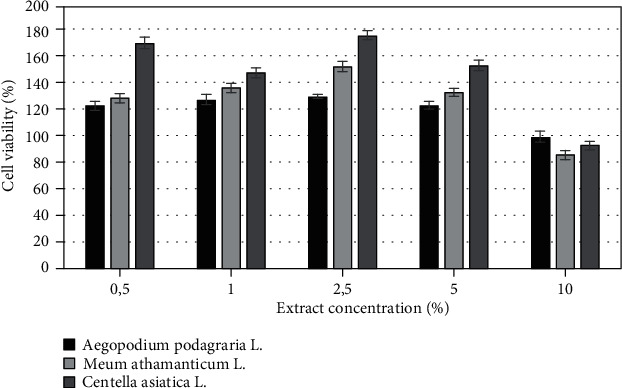
The effect of different concentrations of *Aegopodium podagraria L.*, *Meum athamanticum L.*, and *Centella asiatica L.* extracts (0,5%, 1%, 5%, 10%) on neutral red dye in cultured keratinocytes (A) after 24 h of exposure. Data are the mean ± SD of three independent experiments, each of which consists of three replicates per treatment group.

**Figure 9:**
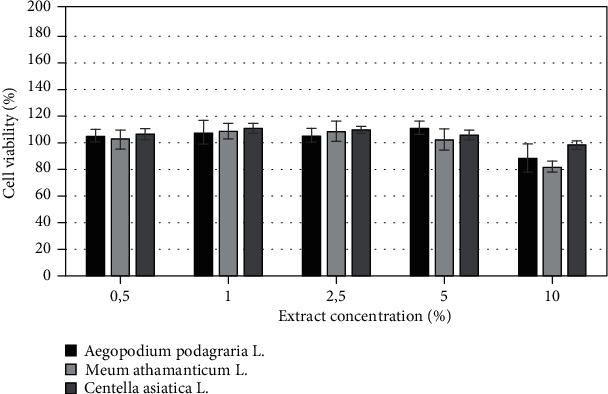
The effect of different concentrations of *Aegopodium podagraria L.*, *Meum athamanticum L.*, and *Centella asiatica L.* extracts (0,5%, 1%, 5%, 10%) on neutral red dye in cultured BJ fibroblasts after 24 h of exposure. Data are the mean ± SD of three independent experiments, each of which consists of three replicates per treatment group.

After application of the extracts in concentrations from 0.5% to 5% on HaCaT cell and BJ cells, a higher increase in HaCat cell proliferation in comparison to the BJ cell line was observed. The research results show that in the case of the BJ line the highest increase in proliferation was observed for *Aegopodium podagraria L.* in a concentration of 5%. This relationship was observed both in the case of the neutral red test and the alamar blue assay. The positive effect of the extract on the examined cell lines can be caused by the presence of compounds such as polyacetylene, in particular, falcarinol and falcarindiol, which play a protective role [18, 64]. In the case of the HaCaT, the highest increase in proliferation was observed when used *Centella asiatica* L. extract in concentration of 2.5% (neutral red test) and also *Centella asiatica* L. extract in concentration of 5% (alamar blue test) (Figures 6-9).

Research conducted by Pitella et al. has shown strong antitumor and antioxidant activity due to the high content of antioxidant compounds, mainly phenolic compounds, in particular flavonoids [64]. Due to its properties, *Centella asiatica* L. regenerates the skin, accelerates healing, and has anti-inflammatory properties [54, 61, 65].

The conducted research shows that extracts obtained from plants of the *Apiaceae* family can be a valuable raw material in the cosmetics industry. Their addition in a concentration of up to 5% has a positive effect on the proliferation of examined skin cells.

In order to examine the potential antiaging properties of the tested extracts, analyses of the activity of two metalloproteinases, collagenase, and elastase were carried out. These enzymes play an important role in physiological processes but are also involved in the etiology of many different skin diseases [66]. Excessive production of reactive oxygen species resulting from internal or external factors increases the synthesis and activates these proteases, leading to degradation of the extracellular matrix, including collagen and elastin fibers [67].

**Figure 10:**
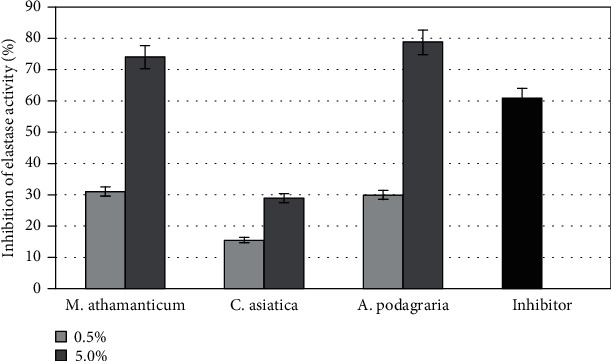
Elastase inhibitory activity of different concentrations (0,5 and 5%) of *Meum athamanticum*, *Centella Asiatica* L., *and Aegopodium podagraria* extracts. Data are the mean of three independent experiments, each consisting of two replicates per treatment group.

In order to evaluate the tested extracts as potential inhibitors of neutrophil elastase, a high throughput in vitro screening test was performed. The principle of this test is to evaluate elastase activity by measuring the hydrolysis of a fluorescent substrate resulting in the release of a fluorescent group that can be detected by fluorometric measurement. The percentage of elastase inhibiting activity in the analyzed extracts is shown in Figure 10. The conducted analyses showed that the tested extracts show different ability to inhibit this enzyme, which results in inhibition of substrate hydrolysis. In order to compare the relative effectiveness of the tested inhibitors, tests with the control inhibitor SPCK were also carried out. The results of the conducted analyses clearly indicate that the tested extracts, especially the *Meum athamanticum* and *Aegopodium podagraria*, show strong inhibition properties of the tested enzyme. 5% concentration of the *A. podagraria* extract inhibited the activity of this enzyme by 79%, while in the case of the *M. athamanticum*, this inhibition for the same concentration was about 74%. The extracts of these plants at a concentration of 0.5% also inhibited elastase activity, while to a much lesser extent of about 30%. In the case of the *C. asiatica*, less inhibition of elastase activity was observed which was 29% for an extract concentration of 5% and 15% for a concentration of 0.5%.

**Figure 11:**
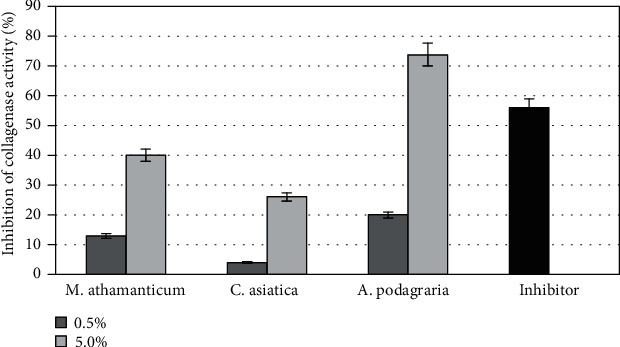
Collagenase inhibitory activity of different concentrations (0,5 and 5%) of *Meum athamanticum*, *Centella Asiatica* L., and *Aegopodium podagraria* extracts. Data are the mean of three independent experiments, each consisting of two replicates per treatment group.

Another method used in the research is a fast and sensitive method enabling the analysis of potential collagenase inhibitors. This method assumes the use of self-quenched BODIPY conjugated to gelatin (type B) as a fluorogenic substrate that can be cleaved by collagenase so that its activity can be evaluated. After proteolytic digestion of the substrate by collagenase, quenched BODIPY emits bright green fluorescence, which is determined by fluorescent measurements. The ability to inhibit the proteolytic activity of collagenase by the analyzed extracts is shown in Figure 11. The obtained test results show that the largest decrease in the produced fluorescent signal indicating inhibition of the activity of the tested enzyme was noted when using the extracts from *A. podagraria* at a concentration of 5%. The use of this concentration resulted in over 70% inhibition of collagenase activity. Gelatin digestion was also reduced in the other two extracts, from *M. athamanticum* and *C. asiatica*, as evidenced by the lack of dequenching of BODIPY but this inhibition is to a much lesser extent. In conducted studies, concentration-dependent enzyme inhibition was observed. Based on the results obtained, it can be stated that especially *A. podagraria* can be seen as a collagenase inhibitor, because it has been shown that it is able (at a concentration of 5%) to inhibit the activity of this metalloproteinase to a greater extent than the commonly known inhibitor of this enzyme (1,10)-Phenanthroline). In order to demonstrate the influence of the studied extracts on the activity of elastase and collagenase, the EC50 value was determined by plotting the log dose-response curve (Table 2). To sum up, the results obtained show that mainly *A. podagraria* extract, but also *M. athamanticum* extract, can be regarded as effective inhibitors of enzymes involved in the degradation of elastin and collagen fibers, which may indicate the legitimacy of their use in the fight against skin aging.

The skin aging process is closely dependent on various pathological and physiological processes, among which the degradation of extracellular matrix biomolecules such as collagen and elastin, which significantly affect the maintenance of good skin condition, is very important [68]. Overexpression of two enzymes from the matrix metalloproteinase class, collagenase and elastase, is closely related to the skin aging process [69]. What is more, literature reports indicate that elastase may stimulate other matrix metalloproteinases, which further accelerate proteolytic degradation of extracellular matrix biomolecules. Elastase has been shown to be able to degrade a wide spectrum of extracellular matrix components such as fibronectin, type IV collagen, proteoglycans, vitronectin, laminin, chondroitin sulfate, and myelin basic protein [70]. It can also stimulate degradation and proteolytic effect by activating cascades of other matrix metalloproteinases [71]. Due to the fact that the results of studies conducted so far by other authors suggest that inhibition of collagenase and elastase is one of the key factors that can prevent the loss of skin elasticity and thereby delay the aging process, as part of this work the possibility of inhibiting these enzymes by extracts from three plants has been examined.

Many authors indicate that secondary metabolites and plant extracts may have collagenase and elastase inhibitory activity [72–77]. This activity is mainly due to the high content of a wide range of various biologically active compounds, such as polyphenols, which include, among others, flavonoids, phenolic acids, tocopherols, and tannins [74]. Literature reports show that plant flavonoids can form complexes and coordinate metal ions due to the presence of carbonyl and hydroxyl groups [66]. This property allows them to bind to metalloenzymes, which include collagenase and elastase, so that these compounds are able to alter or inhibit metabolic pathways [74].

As shown above, our analyses have shown that extracts of *Meum athamanticum L.*, *Aegopodium podagraria L.*, and *Centella asiatica* L. are characterized by a high content of phenolic compounds and flavonoids. As mentioned, these substances may play a role in inhibiting elastase and collagenase. As part of this study, three plants belonging to the *Apiaceae* family were analyzed, of which only one was previously tested for inhibition of these enzymes. Literature data on *C. asiatica L.* anticollagenase and antielastase activity indicate that it has the ability to inhibit these enzymes, but it varies depending on the concentration of the extract used, the type of extraction method, and the extractant. This is probably closely related to biologically active compounds that have been extracted from the plant. The methanol extracts of this plant showed greater inhibition activity of the analyzed enzymes than aqueous extracts [74, 78]. As part of this work, water-glycerin extracts were analyzed, which, compared to analyses carried out by other authors, showed greater inhibition capacity than aqueous extracts, but worse than ethanol extracts. The results presented here indicate that not only *C. asiatica* L. can be seen within this family as a substance with antiaging properties. What is more, it has been demonstrated for the first time that the other two plants belonging to the *Apiaceae* family have better metalloproteinase inhibiting properties and can become a valuable raw material in the cosmetics and pharmaceutical industries. Interestingly, the extract from *A. podagraria L.* in a concentration of 5% showed better inhibitory properties in the enzyme activity analyses than commonly known inhibitors that were used in the study as a control inhibitor. Undoubtedly, a significant role is played by antioxidant compounds, the presence of which has also been confirmed in this work, because as literature data indicate they delay aging processes by scavenging free radicals, which contributes to the inhibition of collagenase and elastase enzymes [74, 79]. This is extremely important because collagen and elastin are responsible for maintaining the structural integrity and elasticity of the skin, and their degradation contributes to the formation of wrinkles and accelerates the skin aging process [66].

The scratch test carried out as part of the research allows real-time assessment of cell movement and morphology as well as measurement of the migration rate of the cells tested. What is more, this test can be carried out on different types of culture plates and is simple, cheap, and easy to analyze [80]. This test is one of the methods that can be used to analyze cell migration, such as keratinocytes or skin fibroblasts, that show collective migration, also known as “sheet migration” [81]. By performing a linear scratch in the cell monolayer with appropriate confluence, it is possible to estimate cell migration and fill the gap [82]. We performed measurements for 24 hours to minimize the role of cell proliferation in filling the gap and limit to assessing the ability of cells to migrate. We also used a lower serum concentration (1%) in the cell medium (serum starvation) compared to the concentration that is used when harvesting keratinocytes and fibroblasts (10%) [80].

Cell migration is an important step in tissue formation and repair. However, researchers have not yet found a clear answer on what influences this process the most. It depends on many factors, such as biochemical communication through paracrine signaling or gap junctions [81]. Their mobility also depends on the strength of adhesion, mechanical flexibility, external migratory signals and cues, dimensionality, and organization of the cellular cytoskeleton [83, 84]. Selective activation of cells at the edge of the wound and cohesion between migrating cells is also important [81, 85]. Some types of cells can also move in groups consisting of cell chains and sheet-like layers [83]. Some authors suggest, however, that coherence and biochemical communication between cells are not necessary for cells to be able to migrate. They explain that the phenomenon of migration is responsible for the spread of cells in the monolayer and release from mechanical constraints, which is associated with the phenomenon of contact inhibition [81].

**Figure 12:**
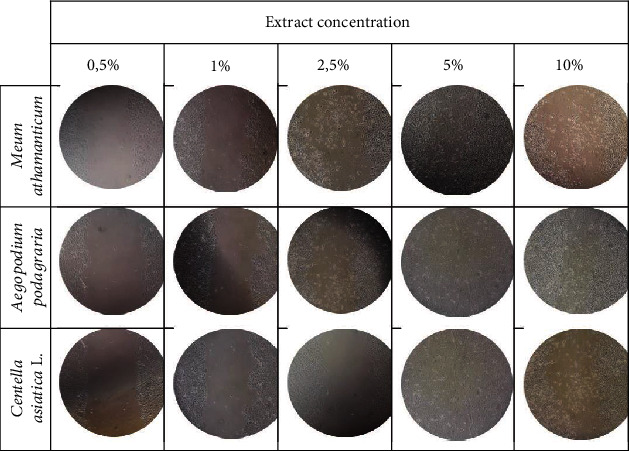
The effect of water-glycerin extracts from *Meum athamanticum L.*, *Aegopodium podagraria L.*, and *Centella asiatica L*. (at a concentration of 0.5-5%) on the migration of HaCaT cells after a 24-hour incubation in a scratch test. A single wound in the middle of the cell monolayer was made using a 10 *μ*l pipette tip. The photo was taken under a magnification of ×10 under a microscope. The analyses were performed in triplicate from three independent experiments.

**Figure 13:**
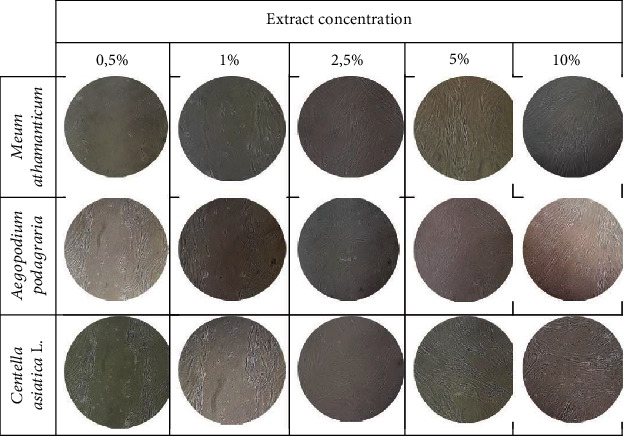
The effect of water-glycerin extracts from *Meum athamanticum L.*, *Aegopodium podagraria L.*, and *Centella asiatica L.* (at a concentration of 0.5-5%) on the migration of fibroblast cells after a 24-hour incubation in a scratch test. A single wound in the middle of the cell monolayer was made using a 10 *μ*l pipette tip. The photo was taken under a magnification of ×10 under a microscope. The analyses were performed in triplicate from three independent experiments.

Proper cell proliferation and migration processes play an important role in maintaining good skin condition and in wound healing [86]. The analyses carried out as part of this work was aimed at assessing the impact of *Meum athamanticum L.*, *Centella asiatica L*., *and Aegopodium podagraria L.* extracts on the migration of keratinocytes and fibroblasts indicate that these plants, depending on the dose used, can stimulate the migration of analyzed cells. The results indicate that the tested extracts after 24 h incubation show similar activity stimulating the migration of both keratinocytes and fibroblasts. Studies have shown that especially 2.5% and 5% have a positive effect on the migration process. Lower concentrations cause much lower induction of cell migration, and the highest concentration used (10%) also turns out to be less effective than concentrations of 2.5 and 5%. In the case of cells not treated with extracts, the migration process was practically imperceptible (Figures 12 and 13).

Wound healing, which often accompanies various skin conditions, is a complex multifactorial mechanism. Many plants have been used in traditional folk medicine for centuries because of their beneficial effects on wound healing. Therefore, *in vitro* testing has so far been carried out using plant extracts or isolated secondary metabolites [87]. In the early stages of wound healing, fibroblasts play an extremely important role, which through active multiplication and migration to the wound area induce the synthesis of a new extracellular matrix and thick actin myofibroblasts [88]. Therefore, the assessment of the migration and proliferative capacity of fibroblasts is crucial in the search for compounds of natural origin that could support wound healing processes.

Although *Centella asiatica* L. is a plant that has been used for a long time in the treatment of wounds and there are a number of scientific studies confirming this, the other two analyzed plants have not yet been tested in this regard. As available data indicate, *C. asiatica L.* is an effective wound healing plant due to the content of many kinds of triterpenoid compounds, mainly including asian acid, madecassic acid, asiaticoside, and madecasoside [84–91]. It has been proved that it shows beneficial effects in the context of wound healing both *in vitro* and *in vivo* [90, 92–94]. This effect is associated, among others, with the increase in collagen type 1 expression [95]. Research also suggests that phytonutrients, especially flavonoids and triterpenoids, also play an important role, because due to their antioxidant properties, they significantly support wound healing [96]. Due to the fact that the analyses carried out as part of this work showed that the analyzed extracts are characterized by high antioxidant capacity, perhaps this scavenging of free radicals is involved in the stimulation of keratinocyte and fibroblast proliferation we have observed [94]. Another possible mechanism is that plant extracts increase fibroblast proliferation, which may be associated with increased collagen production by fibroblasts or fibronectin synthesis [97]. As suggested by studies of other authors, possible mechanisms that can positively affect wound healing and cell migration by *C. asiatica L.* are inhibition of inflammation, promotion of angiogenesis, induction of vasodilatation, and reduction of oxidative stress [94].

It is obvious that further research is needed to better understand the mechanisms responsible for increased migration of cells treated with the extracts tested. However, this work clearly indicates that plants such as *Meum athamanticum L.* or *Aegopodium podagraria L.*, not yet studied for inhibition of matrix metalloproteinase activity and cell migration, can be, like *Centella asiatica* L., effective in the fight against skin aging and significantly affect its regeneration.

## 4. Conclusion

Phenolic compounds and flavonoids from plants are responsible for the antioxidative activities of herbal products. This is explained by their chemical structure and their ability to donate free electrons and hydrogen. The study demonstrated that tested extracts were characterized by a high content of biologically active phenolic compounds and flavonoids. It has been shown that the extracts have an antioxidant potential. The highest antioxidant capacity was observed for the extract of *Aegopodium podagraria L*. In addition, this extract has shown beneficial effects on model skin cells. The results of the conducted analyses clearly indicate that the tested extracts can inhibit the activity of matrix metalloproteinases such as elastase and collagenase. The most promising inhibitor of these enzymes, which can have a significant impact on delaying skin aging processes, turned out to be the extract from *Aegopodium podagraria L*. It has also been shown that the analyzed extracts, especially in concentrations of 2.5 and 5%, have the ability to stimulate the migration of keratinocytes and fibroblasts, which also plays a significant role in maintaining good skin condition and its proper function. These properties may indicate potential use as a valuable ingredient in the cosmetic and pharmaceutical industries as a significant source of a natural antioxidant.

## Figures and Tables

**Table 1:** DPPH radical scavenging activity of water-glycerine extracts from *A. podagraria L.*, *M. athamanticum L.* and *C. asiatica L.* (EC50) each value represents mean ± SD (*n* = 3).

	EC_50_ [%] ± SD
*A. podagraria L*	3, 8 ± 0, 6
*M. athamanticum L*	3, 9 ± 0, 8
*C. asiatica*	4, 2 ± 1, 2

**Table 2:** EC50 values for the *Meum athamanticum*, *Centella asiatica L.*, and *Aegopodium podagraria* demonstrating the effect of the extracts on the activity of elastase and collagenase.

	*Meum athamanticum L.*	*Centella asiatica L.*	*Aegopodium podagraria L.*
	EC_50_ [%] ± SD	EC_50_ [%] ± SD	EC_50_ [%] ± SD
Elastase	0, 92 ± 0, 08	0, 52 ± 0, 04	1, 03 ± 0, 05
Collagenase	1, 89 ± 0, 11	2, 34 ± 0, 08	2, 22 ± 0.07

## Data Availability

The data used to support the findings of this study are available from the corresponding author upon request.
